# Posterior polar annular choroidal dystrophy association with cystoid macular edema

**DOI:** 10.1002/ccr3.1967

**Published:** 2019-01-08

**Authors:** Fernando Del Valle‐Nava, Jorge Sánchez‐Ramos, Angeles Hernández‐Vázquez, Gerardo Gonzalez‐Saldivar, Abel Ramirez‐Estudillo

**Affiliations:** ^1^ Retina Division Fundación Hospital Nuestra Señora de la Luz México City México; ^2^ Retina Division St. Michael’s Hospital Toronto Ontario Canada

**Keywords:** choroidal dystrophies, cystoid macular edema, posterior polar annular choroidal dystrophy, retinal pigment epithelium atrophy

## Abstract

Posterior polar annular choroidal dystrophy (PPACD) is an uncommon retinal dystrophy causing nyctalopia. PPACD has been characteristically described as a foveal sparing dystrophy. We report the first case with cystoid macular edema association.

## 1 CASE

Posterior polar annular choroidal dystrophy (PPACD) is an uncommon disease characterized by retinal pigment epithelium and choriocapillaris atrophy in an annular pattern surrounding the optic nerve and temporal vascular arcades sparing the fovea.^1^ There are very few reports about this entity; however, there are no reports with macular edema association.

**Figure 1:**
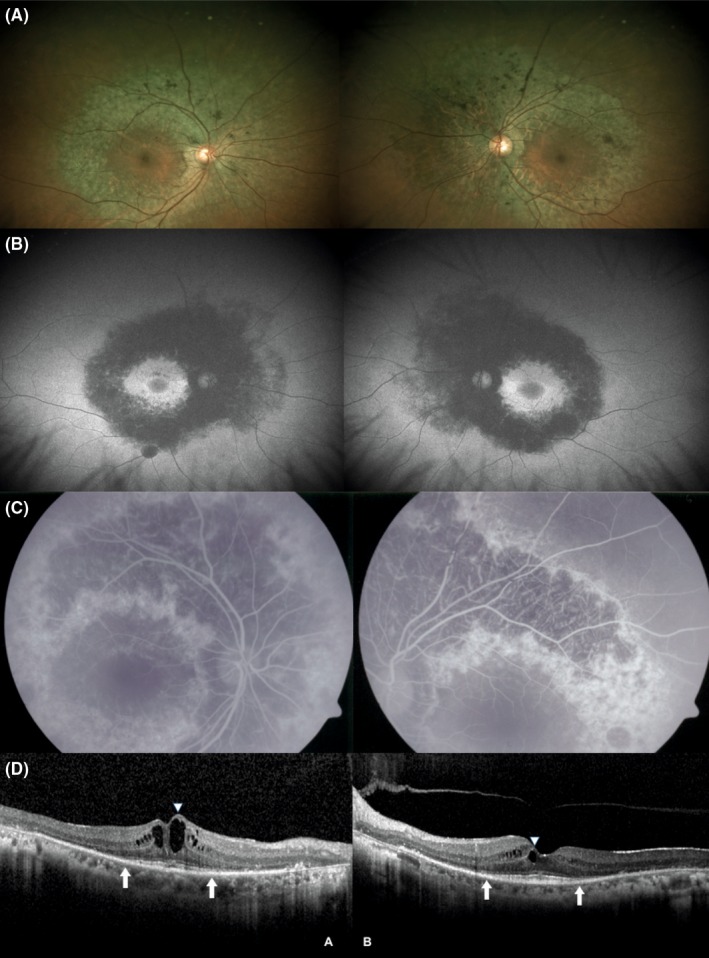
A, Fundus photographs show bilateral RPE and choroidal atrophy around the optic nerve and temporal vascular arcades with foveal sparing. B, Fundus autofluorescence displays bilateral annular hypoautofluorescence corresponding to the areas of RPE atrophy. C, Fluorescein angiography demonstrates an annular hypofluorescence defect due to the lack of filling of both retinal and choroidal vasculatures, which correspond to chorioretinal atrophy. D, SD‐OCT illustrates intraretinal cystoid spaces on the inner nuclear layer as well as parafoveal atrophy of choriocapillaris and outer retinal layers (arrows)

A 65‐year‐old woman presented with a 3‐year history of nyctalopia. On referral, visual acuity was 20/25 in the right eye and 20/20 in the left eye. Anterior segment examination for both eyes was normal. Bilateral fundus examination revealed retinal pigment epithelium (RPE) and choriocapillaris atrophy in an annular pattern along the optic nerve and the temporal vascular arcades, sparing the central macula and peripheral retina (Figure 1A).

Fundus autofluorescence imaging displayed a well‐defined annular pattern of hypoautofluorescence concentric to the parafoveal area, corresponding to the region of atrophy (Figure 1B). Retinal fluorescein angiography (FA) demonstrates an annular hypofluorescence defect due to the lack of filling of both retinal and choroidal vasculatures corresponding to the atrophy region (Figure 1C). Spectral‐domain optical coherence tomography (SD‐OCT) showed cystoid macular edema with outer retinal atrophy sparing subfoveal region (Figure 1D). Electroretinography revealed a depressed cone response with a normal rod response. Due to the clinical imaging and electrophysiological studies, diagnosis of posterior polar annular choroidal dystrophy (PPACD) was made.^1^, ^2^

Posterior polar annular choroidal dystrophy is a rare disease with a characteristic pattern of outer retinal and choroidal atrophy in an annular form along the optic nerve and the temporal vascular arcades, with central and peripheral retina sparing.^1^, ^2^ Although cystoid macular edema has characteristically been described as a concomitant pathology in other retinal dystrophies, it has never been associated with PPACD.

## CONFLICT OF INTEREST

None declared.

## AUTHOR CONTRIBUTION

FDV, JSR, and GGS: wrote and prepared the manuscript. AHV: interpreted the electroretinography. ARE: revised the manuscript and gave the final approval.
